# HTLV-1 and -2 envelope SU subdomains and critical determinants in receptor binding

**DOI:** 10.1186/1742-4690-1-41

**Published:** 2004-12-02

**Authors:** Felix J Kim, Nicolas Manel, Edith N Garrido, Carine Valle, Marc Sitbon, Jean-Luc Battini

**Affiliations:** 1 Institut de Génétique Moléculaire de Montpellier (IGMM), CNRS-UMR5535, IFR122 1919 Rte de Mende, F-34293 Montpellier Cedex 5, France; 2 Current address: Memorial Sloan-Kettering Cancer Center 1275 York Ave, New York, NY, 10021, USA

## Abstract

**Background:**

Human T-cell leukemia virus (HTLV) -1 and -2 are deltaretroviruses that infect a wide range of cells. Glut1, the major vertebrate glucose transporter, has been shown to be the HTLV Env receptor. While it is well established that the extracellular surface component (SU) of the HTLV envelope glycoprotein (Env) harbors all of the determinants of interaction with the receptor, identification of SU subdomains that are necessary and sufficient for interaction with the receptor, as well as critical amino acids therein, remain to be precisely defined. Although highly divergent in the rest of their genomes, HTLV and murine leukemia virus (MLV) Env appear to be related and based on homologous motifs between the HTLV and MLV SU, we derived chimeric HTLV/MLV Env and soluble HTLV-1 and -2 truncated amino terminal SU subdomains.

**Results:**

Using these SU constructs, we found that the 183 and 178 amino terminal residues of the HTLV-1 and -2 Env, respectively, were sufficient to efficiently bind target cells of different species. Binding resulted from *bona fide *interaction with the HTLV receptor as isolated SU subdomains specifically interfered with HTLV Env-mediated binding, cell fusion, and cell-free as well as cell-to-cell infection. Therefore, the HTLV receptor-binding domain (RBD) lies in the amino terminus of the SU, immediately upstream of a central immunodominant proline rich region (Env residues 180 to 205), that we show to be dispensible for receptor-binding and interference. Moreover, we identified a highly conserved tyrosine residue at position 114 of HTLV-1 Env, Tyr_114_, as critical for receptor-binding and subsequent interference to cell-to-cell fusion and infection. Finally, we observed that residues in the vicinity of Tyr_114 _have lesser impact on receptor binding and had various efficiency in interference to post-binding events.

**Conclusions:**

The first 160 residues of the HTLV-1 and -2 mature cleaved SU fold as autonomous domains that contain all the determinants required for binding the HTLV receptor.

## Background

Human T-cell leukemia virus type 1 (HTLV-1) has been found primarily in CD4+ and CD8+ T-lymphocytes *in vivo *[1-3], whereas CD8+ T-lymphocytes are thought to be the *in vivo *reservoir of HTLV-2 [4]. However, the *in vitro *tropism of HTLV-1 and -2, as determined using HTLV envelope-pseudotyped virions or envelope-induced cell fusion assays, appears to be ubiquitous [5-7]. Indeed, we recently showed that Glut1, the ubiquitous vertebrate glucose transporter, serves as a receptor for HTLV-1 and -2 envelope glycoprotein (Env) [8]. While the precise organization and properties of the receptor-interacting Env domains has not been reported, we found that the amino terminal two-thirds of the HTLV-1 extracellular surface component (SU) are sufficient to confer HTLV-1 tropism to an ecotropic Friend murine leukemia virus (F-MLV) Env [9]. A cell fusion interference assay performed with this HTLV/F-MLV Env chimera and the parental Env confirmed that this 215 amino acid Env domain, harbors HTLV-1 receptor-binding determinants [9].

**Figure 1:**
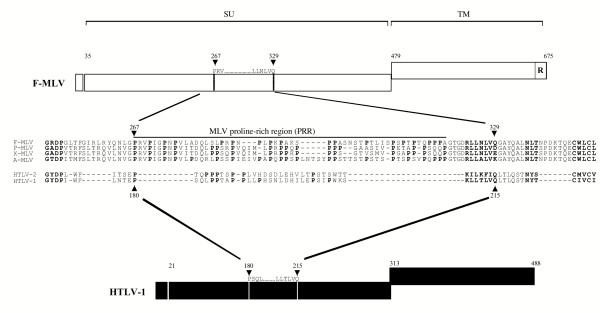
**Homologous modular domains in HTLV and MLV envelopes. **Friend-MLV (F-MLV) Env and HTLV-1 Env are schematically represented as open and solid boxes, respectively. Boxes represent, from left to right, the signal peptide which comprises the first 34 and 20 amino acid residues of F-MLV and HTLV Env, respectively, the extracellular surface component (SU) and the transmembrane component (TM) including the carboxy terminal R peptide in F-MLV, which is cleaved in the mature Env glycoprotein [64, 65]. Env landmark positions are indicated and the MLV proline-rich regions (PRR) and the HTLV SU PRR homologue (PRRH) are delineated by vertical lines within the SU at the positions indicated by solid arrowheads. The PRR and PRRH start at the first proline (P) residue downstream of the conserved GXDP motif. Env sequences represented in the figure are obtained from F-MLV strain 57 (accession number CAA26561); P-MLV, F-MCF polytropic MLV (AAA46483); X-MLV, NZB xenotropic MLV (AAA46531); A-MLV, amphotropic MLV strain 4070A (AAA46515); HTLV-2 (NP_041006); and HTLV-1, MT2 strain (VCLJMT). Residue numbering starts from the first methionine of the Env signal peptides. Proline residues and homologous motifs are noted in bold. Amino acid sequence alignments were performed using the Clustal program in the Megalign alignment software package (DNAStar) with manual adjustments.

The corresponding domain in MLV Env SU – located upstream of a conserved K/R L L T/N L V Q motif in the SU of the HTLV-1 and F-MLV Env [9,10] – is well characterized and comprises two main functional regions: an amino terminal sequence harboring the receptor-binding determinants, VRA, VRB and VRC [11-13], and a proline-rich region (PRR), starting at the first proline residue of the GPRVPIGP sequence [11,14] and flanked by two highly conserved GXDP [15] and CXXC [16] motifs (Figure 1). In the ecotropic and amphotropic (Ampho) MLV Env, the PRR is a putative hinge region implicated in conformational changes, triggered after receptor binding, and subsequent fusion [17,18]. In the central region of the HTLV SU, a short sequence (Env residues 180 to 205) harbors high proline content and could be a homologue of the MLV PRR.

Several studies using synthetic peptides and neutralizing antibodies against the HTLV Env have shown that determinants within this proline rich region homologue (PRRH) are involved in interference to Env-mediated syncytium formation [19-21]. The PRRH had been thought to encode the receptor-binding domain, as based on cell-to-cell fusion assays [19,22-24]. However, although PRRH synthetic peptides can block HTLV Env-mediated syncytia formation, they have no effect on HTLV SU binding [25] and infection [26]. Indeed, we and others have shown that Env receptor binding *per se*, as well as interference to receptor-binding, cell-to-cell fusion, syncytium formation, and infection involve several distinct cell surface-associated parameters [27-29]. In the present report, we produced soluble forms of wild-type and mutant HTLV-1 and 2 SU amino terminal subdomains and tested their receptor-binding abilities. We also tested their ability to specifically interfere with HTLV Env cell surface binding, Env-mediated cell-to-cell fusion, and retroviral infection. By testing these essential parameters of Env-mediated dissemination, we delineated the Env receptor-binding domain (RBD) to the first 160 residues of the mature HTLV-1 and -2 SU, excluding the PRRH, and we identified a conserved tyrosine residue at position 114 of HTLV-1 Env as a critical determinant for HTLV Env receptor binding.

## Results

### Motif conservation and similar modular organization of HTLV and MLV SU, and identification of a proline-rich region homologue (PRRH) in the HTLV SU

As shown in Figure 1, our alignment of the MLV and HTLV SU reveals several notable motif conservations outlining a similar modular organization of the MLV SU and HTLV SU. A (K/R)LL(T/N)LVQ motif, highly conserved between the F-MLV and HTLV-1 SU, is located immediately downstream of the PRR and its PRRH counterpart, respectively. Another highly conserved motif between MLV and HTLV, GXDP, is found immediately upstream of the PRR/PRRH (Figure 1). These two motifs compelled us to notice the PRRH, between the PSQ and KLLTLVQ sequences in HTLV-1, and between the PTQ and KILKFIQ sequences in HTLV-2 (Figure 1). As counted from the first and last proline in the delineated sequence, the PRRH has a proline content of 30.8% and 30.4% for HTLV-1 and -2, respectively. This is slightly lower than the 35.3%, 36%, 36%, and 35.6% proline content for the ecotropic, polytropic, xenotropic, and amphotropic MLV Env, respectively (Figure 1). The presence of a PRRH in the HTLV SU appeared to be characteristic of their MLV-like modular organization, since HTLV SU average proline content outside of the PRRH does not exceed 11%.

### Functional, soluble HTLV Env-receptor binding determinants

**Figure 2:**
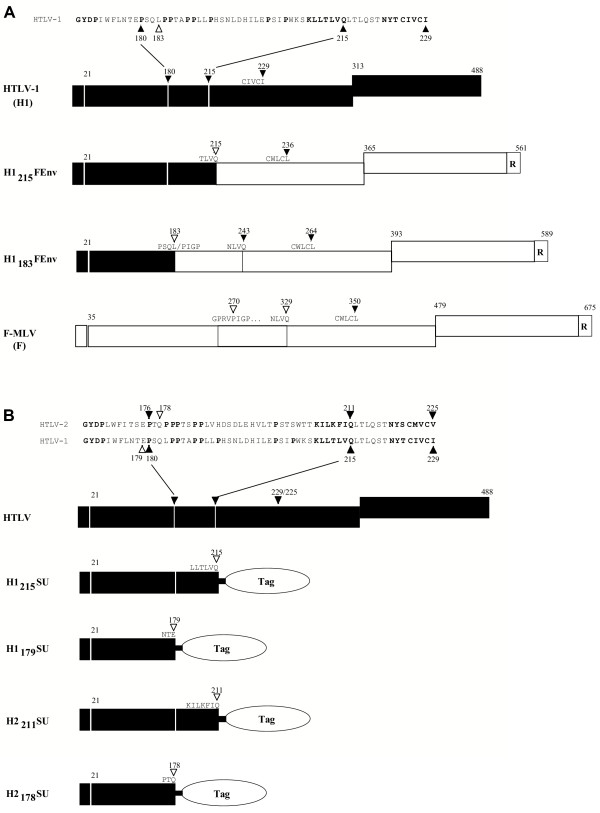
**Schematic representation of HTLV/MLV Env chimeras and HTLV SU amino terminal subdomains. **Env landmark positions are indicated and SU landmark sequences and positions are indicated by arrowheads. Open arrowheads indicate the position of construct borders. (A) HTLV/MLV Env chimeras. The H1_215_FEnv and H1_183_FEnv HTLV/MLV Env chimeras were obtained by replacing the 329 and 269 amino terminal residues of the F-MLV Env (open boxes) with the amino terminal 215 and 183 amino acid residues of the HTLV-1 Env (solid boxes), respectively. The H1_215_FEnv chimera, previously described and formerly designated HHproFc [9], has been renamed here for sake of nomenclature homogeneity. (B) Soluble HTLV-1 (H1) and HTLV-2 (H2) SU amino terminal subdomains, H1_215_SU, H2_211_SU, H1_179_SU, and H2_178_SU were constructed as fusion proteins with a carboxy terminal hemagglutinin (HA) or rabbit immunoglobulin Fc (rFc) tag. All amino acid residue numbering starts from the first methionine of the HTLV-1 or -2 Env signal peptide, the amino terminal 20 and 21 aa residues, respectively.

**Figure 3:**
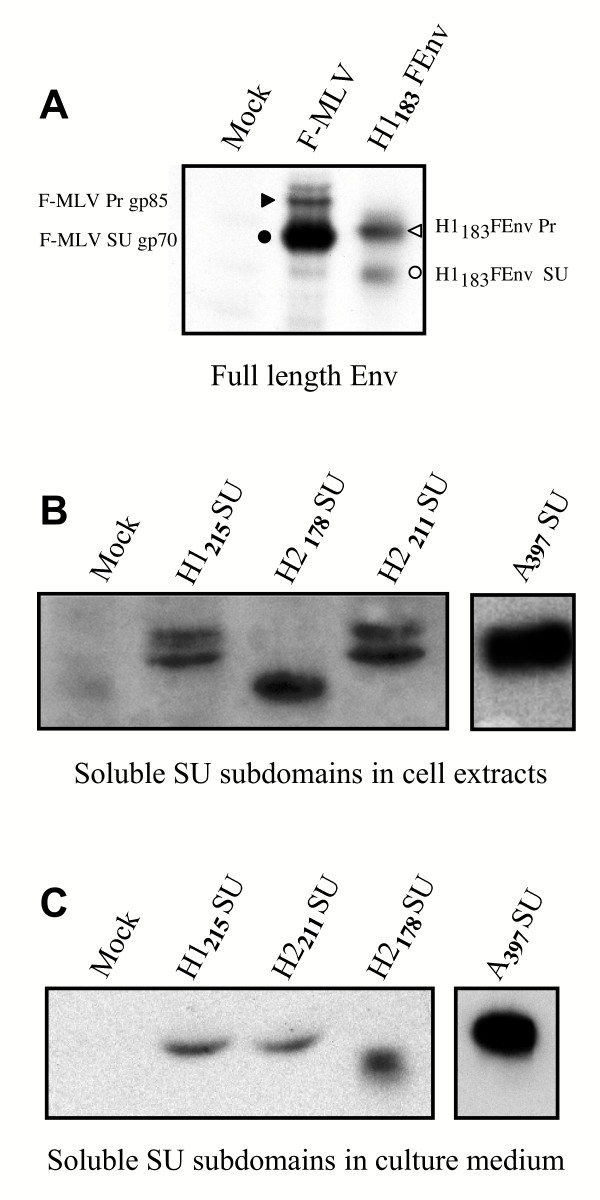
**Intracellular expression of HTLV-1 Env chimeras and soluble SU subdomains. **Cell extracts (A, B) or culture supernatants (C) were prepared from 293T cells transfected with either full length Env (A) or soluble SU subdomains (B, C) expression vectors as depicted in figure 2. Membranes were probed with either (A) an anti-MLV SU antiserum to detect F-MLV and H1_183_FEnv uncleaved Env precursor proteins (F-MLV Prgp85 and H1_183_Fenv Pr, respectively) indicated by arrowheads, and cleaved SU (F-MLV SUgp70 and H1_183_FEnv SU, respectively) indicated by circles, or (B, C) an anti-rabbit IgG antiserum to detect carboxy terminal rFc-tagged soluble subdomains, including the Ampho-MLV SU subdomain (A_397_SU).

MLV SU receptor binding determinants are all located upstream of the PRR [11,30]. To test whether the HTLV Env receptor binding determinants are also located upstream of the potential PRRH, we constructed a chimeric Env and several soluble HTLV-1 and -2 SU amino terminal subdomains. The chimeric HTLV/MLV Env, H1_183_FEnv, comprises the 183 amino terminal residues of the HTLV-1 SU ending with the PSQL residues fused to the PIGP sequence of the F-MLV PRR (Figure 2A). In this Env chimera the receptor-binding domain (first 269 residues) of the F-MLV Env was replaced with the potentially corresponding domain of the HTLV-1 Env SU (Figure 2A). The chimeric H1_183_FEnv construct – which lacks the HTLV PRRH but has the MLV PRR – was properly expressed in transfected cells and was revealed on immunoblots with an anti-MLV SU polyclonal antibody (Figure 3A). Accordingly, an anti-HTLV-1 monoclonal antibody raised against a PRRH epitope did not bind this chimeric Env (data not shown).

HTLV-1 and -2 SU amino terminal subdomains with or without their respective PRRH were constructed as fusion proteins with either an influenza hemagglutinin (HA) or rabbit immunoglobulin Fc (rFc) carboxy terminal tag (Figure 2B). The H1_215_SU and H2_211_SU subdomains comprise the first 215 and 211 residues, counting from the first methionine in the signal peptide through the KLLTLVQ of HTLV-1 and KILKFIQ of HTLV-2 Env, respectively (Figure 2B). The H1_179_SU and H2_178_SU, comprising the amino terminal 179 and 178 amino acids of the HTLV-1 and -2 Env, respectively, exclude the PRRH sequence (Figure 2B).

Cell lysates and cell culture supernatants were analyzed to evaluate intracellular expression and secretion of functional SU amino terminal domains in transfected-cell cultures, respectively. H1_215_SU and H2_211_SU, containing the PRRH sequence, and H2_178_SU lacking this PRRH were all efficiently expressed in transfected cells (Figure 3B). It is noteworthy, however, that recovery of tagged H1_179_SU molecules was largely inefficient because the vast majority of this protein was cleaved (data not shown). In contrast, no significant cleavage was observed with the other soluble domains released in the medium (not shown) (Figure 3C). As expected for immunoadhesins, H1_215_SU, H2_211_SU, and H2_178_SU rFc-tagged domains were detected as dimers under non-reducing conditions (not shown). Immunoblots of cell extracts revealed two forms of intracellular H1_215_SU and H2_211_SU (Figure 3B); this was likely due to variable glycosylation of these subdomains. However, a single secreted, soluble form of each of these amino terminal subdomains was detected in cell culture supernatants (Figure 3C).

A truncated Ampho-MLV SU-rFc fusion protein that comprises the amino terminal 397 residues of the Ampho-MLV Env fused to a carboxy terminal rFc tag was constructed (A_397_SU) and used as a heterologous control. A single form of this truncated SU was efficiently expressed in transfected cells (Figure 3B), and abundantly secreted in cell culture medium (Figure 3C).

### HTLV-1 and -2 SU subdomains with HTLV receptor binding properties

**Figure 4:**
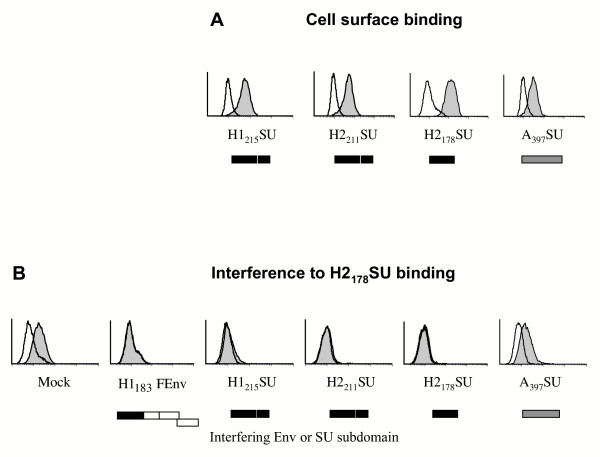
**HTLV-1 and -2 SU subdomains interfere with HTLV Env SU cell surface binding. **(A) Conditioned medium from control 293T cells (open histograms) or from 293T cells expressing soluble rFc-tagged HTLV-1 H1_215_SU, HTLV-2 H2_211_SU and H2_178_SU, or Ampho-MLV A_397_SU subdomains (filled histograms), were incubated with A23 hamster cells for 30' at 37°C and binding was assessed by flow cytometry following addition of a secondary FITC-conjugated anti rabbit IgG antibody. Similar results were obtained in binding assays performed using all cell lines described in the text. (B) To assess binding interference, target 293T cells were transfected with the indicated Env construct and subsequently incubated with the HA-tagged H2_178_SU domain (filled histograms). Binding was detected by FACS following incubation with an anti HA 12CA5 mouse mAb and a FITC-conjugated anti mouse IgG antibody. Open histograms represent background levels of fluorescence. SU constructs are schematically represented below each graph by solid (HTLV), open (F-MLV) or grey (Ampho-MLV) boxes.

The amino terminal subdomains were tested for their ability to bind to HTLV receptor-presenting cells by flow cytometry. Using this cell surface binding assay, all of the soluble HTLV SU subdomains bound to the A23 hamster fibroblast cell line (Figure 4) as well as to all other cell lines tested, including 293T (human kidney fibroblasts), NIH3T3 and NIH3T3TK^- ^(murine fibroblasts) [29], HeLa (human ovarian carcinoma cells), D17 (canine fibroblast), Jurkat (suspension human T cell line), activated primary human T cells, and numerous other cell lines and primary cell types that are thought to express the HTLV receptor. As expected from our previous work [31], none of these soluble HTLV SU subdomains showed detectable binding on resting T lymphocytes. Notably, binding of the HTLV SU to these cells occurred whether they formed or not syncytia in the presence of HTLV Env [29] and data not shown). Binding by H2_178_SU was similar to H2_211_SU, demonstrating that the first 158 residues of the mature HTLV-2 SU, without the 20 amino acids of the amino terminal signal peptide, are sufficient for cell surface binding, and therefore that the PRRH is not required for receptor binding (Figure 4A).

To determine whether cell surface binding of these soluble SU domains corresponded to *bona fide *binding to the HTLV receptor, we performed an Env-specific binding interference assay. In this assay, transfection of the above described chimeric Env and SU subdomains into 293T cells resulted in interference to cell surface binding by the soluble HA-tagged H2_178_SU subdomain (Figure 4B). Indeed, nearly complete interference was observed when cells were transfected with the amino terminal subdomain constructs, in the presence and absence of PRRH sequences (H1_215_SU and H2_211_SU versus H1_183_FEnv and H2_178_SU) (Figure 4B). This effect was specific as HTLV SU binding was not inhibited by a heterologous A_397_SU domain (Figure 4B). Therefore, we showed that the first 163 and 158 residues, with a cleaved signal peptide, of the mature HTLV-1 and HTLV-2 SU, respectively, contained the entire HTLV Env RBD. These data also showed that HTLV-1 and 2 cross-interfered, consistent with the fact that they recognize the same cell surface receptor for infection [8,32].

### Interference to HTLV Env-mediated cell-to-cell fusion by HTLV SU amino terminal subdomains

Viral envelope interference occurs when cell surface receptors are occupied by receptor-interacting Env components [33-35]. Since interference to the different Env-mediated functions involves distinct components [27-29], we also tested the abilities of the H1_183_FEnv and the HTLV SU amino terminal subdomains to interfere with HTLV Env-mediated cell fusion. Interference to cell fusion was measured using a quantitative HTLV envelope cell fusion interference assay (CFIA), as previously described [9].

**Figure 5:**
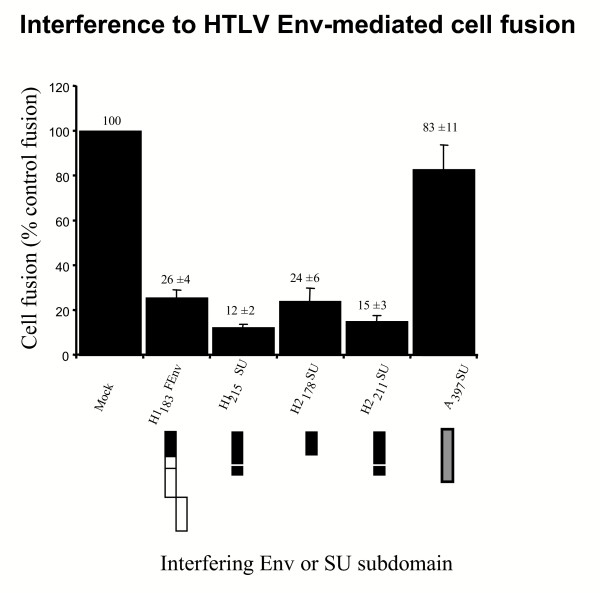
**HTLV-1 and -2 SU subdomains interfere with HTLV Env-mediated cell fusion. **Cell-to-cell fusion assays were performed by cocultivating fusogenic HTLV-1 Env-expressing cells with target cells expressing the Env derivatives indicated and schematically represented below each histogram. HTLV-1 Env-mediated cell fusion in the presence of target cells transfected with empty vector (Mock) yielded 200 to 1000 blue foci in 4 independent experiments and these levels were defined as 100% cell fusion. Cell fusion levels in the presence of HLTV SU mutants or the A_397_SU control Ampho-MLV SU subdomain is shown as percent of control. Mean fusion percentages were determined from three to four independent experiments. Error bars represent the standard error of the mean.

HTLV-1 Env-induced cell fusion was significantly diminished upon expression of the H1_215_SU subdomain in target cells, 12% ± 2% of control fusion (*P *< 0.001), consistent with previous observations using the H1_215_FEnv chimera [9]. Significant interference to cell fusion was also observed with the H1_183_FEnv chimera, which lacked a PRRH, down to 26% ± 4% of control fusion (*P *< 0.001) (Figure 5). The corresponding HTLV-2 SU subdomains produced a nearly identical cell fusion interference profile: interference by the H2_211_SU isolated domain, in which the PRRH was maintained, resulted in 15% ± 3% of control cell fusion levels, while the H2_178_SU subdomain, lacking the HTLV PRRH, inhibited HTLV-1 Env-induced cell fusion to 24% ± 6% of control levels (*P *< 0.001) (Figure 5). It is noteworthy that similar data were obtained when comparing cell fusion interference by H1_215_FEnv and H1_183_FEnv. These effects were specific to HTLV SU amino terminal domains as A_397_SU did not interfere with HTLV-1 Env-mediated cell fusion (83% ± 11% of control fusion) (Figure 5). Furthermore, no interference was observed when these truncated HTLV SU fragments and chimeric Env were tested against heterologous, fusogenic control Env such as AΔR Env, FΔR, XenoΔR and VSVG (data not shown). Altogether, these results confirmed our findings that receptor-binding determinants are present within the first 183 and 178 amino acids of the HTLV-1 and -2 Env, respectively. They also indicated that the PRRH (H1_215_SU and H2_211_SU), although unnecessary for receptor binding, modulates the efficiency of interference to HTLV Env-induced cell-to-cell fusion (*P *< 0.03).

### Interference to HTLV Env-mediated infection by HTLV SU amino terminal subdomains

Interference, as described above, was based on the inhibition of cell-to-cell fusion induced by fusogenic Env expressed in the absence of other viral proteins. We further evaluated the abilities of the Env chimeras and soluble subdomains to specifically interfere with HTLV Env-mediated infection. HTLV Env-pseudotyped MLV virions, MLV(HTLV), were produced to infect 293T target cells. Because these recombinant cell-free virions are not competent for replication, this viral pseudotype infection assay tests a single round of infection, and does not measure replication and subsequent exponential viral dissemination. Therefore, relative infection values are expressed in linear rather than logarithmic scales.

**Figure 6:**
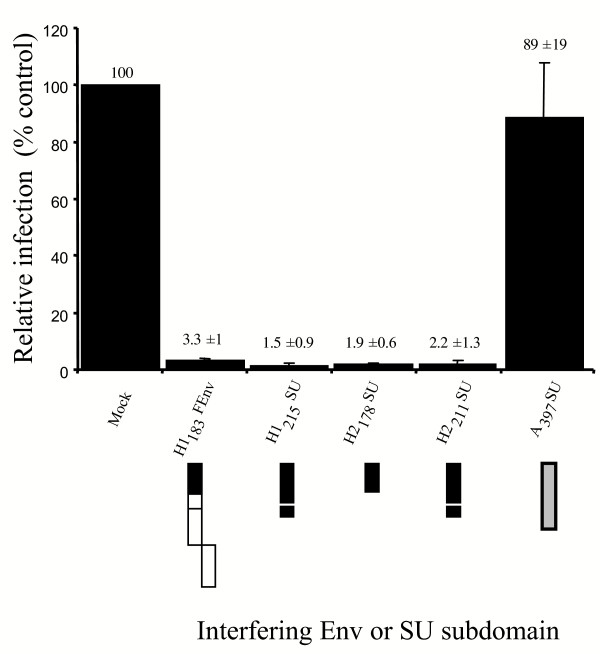
**HTLV-1 and -2 SU subdomains interfere with infection by HTLV envelope-pseudotyped virions. **293T cells (5 × 10^5^) expressing the indicated interfering Env derivatives were infected with cell-free HTLV-2 Env-pseudotyped virions MLV(HTLV) carrying a LacZ reporter gene. Infected cells were detected 2 days later by X-gal staining. Infection values are represented as percent of control infection, i.e., relative to infection of mock (pCDNA3.1) transfected target cells, calculated as infectious units per ml of virus containing supernatant (i.u./ml). Data are representative of at least three independent experiments performed in duplicate. Error bars represent the standard error of the mean.

Infection of mock-transfected target cells, devoid of interfering Env domains, resulted in a mean infection value of 9905 ± 1117 infectious units per ml (iu/ml), and this was taken as 100% control infection (Figure 6). Similar values, 8803 ± 1871 iu/ml or 89% ± 19% of control infection, were obtained upon infection of target cells expressing a heterologous SU subdomain, A_397_SU (Figure 6). Expression of the H1_183_FEnv and H1_215_FEnv chimeric Env in target cells significantly reduced MLV(HTLV) infection to 324 ± 98 iu/ml, 3.3% ± 1% of control infection, and to 307 ± 129 iu/ml, 3.1% ± 1.3% of control infection, respectively (Figure 6 and data not shown). Similarly, the H2_178_SU and H2_211_SU subdomains diminished MLV(HTLV) infection to 191 ± 56 iu/ml and 215 ± 122 iu/ml, 1.9% ± 0.6% and 2.2% ± 1.3% of control infection, respectively (Figure 6). The specificity of interference to infection by HTLV Env constructs was assessed by their lack of interference abilities toward Ampho-MLV Env-pseudotyped virions, MLV(Ampho) (data not shown). Thus, for both HTLV-1 and -2, the amino terminal domain upstream of the PRRH was sufficient for specific interference to HTLV Env-mediated infection. Furthermore, in contrast to the cell fusion interference assays described above, the PRRH did not detectably influence MLV(HTLV) infection.

Because HTLV dissemination appears to occur mostly via cell-to-cell contact, we also tested envelope interference to infection by HTLV-1 SU amino terminal domains using a cell-to-cell transmission interference assay. In this assay, cells harboring interfering chimeric Env and soluble subdomains were cocultured with cells producing MLV(HTLV) virions. Transfection of either chimeric Env or soluble subdomains into HeLa target cells decreased MLV(HTLV) infection to levels similar to those observed in the cell fusion interference assay presented in figure 5 (data not shown).

### Identification of residues within the HTLV SU amino terminal domain that modulate receptor binding and HTLV Env-mediated interference

**Figure 7:**
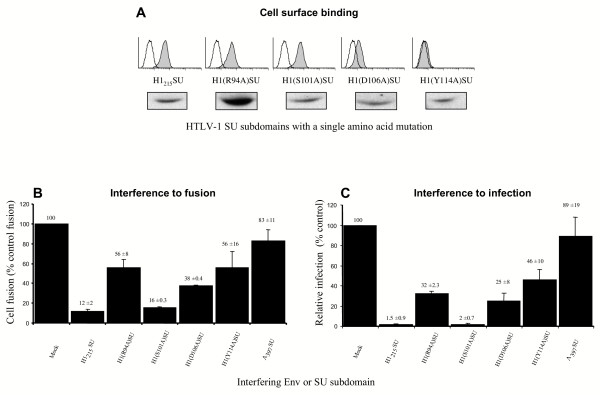
**HTLV-1 SU amino terminal domain mutants. **(A) H1_215_SU constructs were generated with the following SU amino terminal point mutations; R94A, S101A, D106A and Y114A. The abilities of these soluble H1_215_SU constructs to bind 293T cells were assessed by flow cytometry (gray histograms). The levels of expression of the various soluble SU subdomains are shown under each histogram. The abilities of the H1_215_SU mutants to interfere with (B) HTLV Env-induced cell fusion and (C) MLV(HTLV) pseudotype infection was assayed as described in Figs. 5 and 6. Data are representative of at least three independent experiments performed in duplicate. Error bars represent the standard error of the mean.

Two key residues contained in the HTLV SU RBD and conserved between HTLV-1 and -2, arginine 94 (Arg_94_) and serine 101 (Ser_101_) for HTLV-1 Env which correspond to Arg_90_and Ser_97 _in HTLV-2 Env, have been shown to alter cell-to-cell fusion and infection when mutated [36,37]. To determine whether mutations of these residues had an effect on receptor binding, we generated H1_215_SU subdomains with either Arg_94 _or Ser_101 _mutated to Ala, yielding the mutant H1(R94A)SU and H1(S101A)SU subdomains, respectively. We also evaluated mutations of Asp_106_, mutant H1(D106A)SU, and Tyr_114_, mutant H1(Y114A)SU, both residues found to be highly conserved between all human and simian T cell leukemia viruses (unpublished observations). Surprisingly, cell surface binding profiles of H1(R94A)SU and H1(S101A)SU mutants were not significantly altered when compared to binding by the parental H1_215_SU, whereas the H1(D106A)SU mutant presented reduced binding to HTLV receptor-bearing cells and the H1(Y114A)SU mutant showed a nearly complete abrogation of cell surface binding (Figure 7A). Loss of binding observed with the two latter mutants was not due to decreased soluble SU fragment production, as assessed by immunoblotting of transfected-cell culture media (Figure 7A). Moreover, equivalent binding profiles were obtained when the same mutations were introduced into the HTLV-2 soluble RBD H2_178_SU (data not shown). Altogether, these experiments demonstrated that Tyr_114_, and to a lesser extent Asp_106_, are key residues involved in HTLV Env receptor binding.

We next tested the abilities of these mutants to interfere with HTLV Env-mediated cell fusion and infection, using the assays described above. As mentioned above, all wild-type and mutant HTLV SU subdomains were produced and secreted with a similar efficiency (Figure 7A). Expression of the H1(D106A)SU and H1(Y114A)SU mutants, with decreased capacities to bind the HTLV receptor, correlated with decreased interference to HTLV Env-mediated cell fusion and infection. Indeed, H1(Y114A)SU, which had nearly undetectable level of binding, showed the lowest levels of interference and thus allowed the highest levels of HTLV Env-mediated cell fusion and infection (56% ± 16% and 46% ± 10%, respectively) (Figure 7). Nevertheless, levels of fusion and infection were lower than that observed when the heterologous A_397_SU was used as a negative control of interference (83% ± 11% and 89% ± 19% for cell fusion and infection, respectively). Thus, overexpression of mutant HTLV SU fragments with highly decreased receptor binding abilities can still exert, albeit to a significantly lesser extent, interference to HTLV Env-mediated cell fusion and infection.

We found that similar levels of interference to HTLV Env-mediated cell fusion and infection were observed when either the parental H1_215_SU or the mutant H1(S101A)SU were expressed in target cells (Figure 7B and 7C). This is consistent with the capacity of this mutant to bind target cells at levels similar to that of wild type H1_215_SU. However, interference to HTLV Env-mediated cell fusion and infection did not always correlate with cell surface binding profiles. While the H1(R94A)SU mutant inhibited cell fusion and infection, its effects were significantly lower than those of the wild-type H1_215_SU (56% ± 8% and 32% ± 2.3%, respectively) (Figure 7B,7C). Thus, although neither Arg_94 _nor Ser_101 _of the HTLV-1 SU appears to play a direct role in binding, Arg_94 _modulates HTLV Env-mediated fusion and infection (Figure 7), likely via post-binding effects rather than binding *per se*. In conclusion, Tyr114 appeared as the main determinant identified so far for HTLV Env binding, whereas the effects previously described with Arg_94 _and Ser_101 _are most likely associated with post-binding events.

## Discussion

Here, we report the generation of MLV Env with chimeric HTLV/MLV SU and truncated HTLV-1 and -2 amino terminal SU subdomains that can be expressed in and secreted from eukaryotic cell lines in functional, soluble form. Using these constructs, we demonstrated that the amino terminal 163 and 158 residues (i.e., expunged of their Env signal peptide) of the mature HTLV-1 and -2 Env SU, respectively, were sufficient to exert both HTLV receptor binding and efficient interference to diverse HTLV Env-mediated functions, including binding, cell-to-cell fusion and cell-free as well as cell-to-cell infection. Although the PRRH sequence comprising amino acid residues 180 to 215 of the HTLV-1 Env and 176 to 211 of the HTLV-2 Env was previously thought to be a receptor binding site, our data preclude a major role for this region in the binding properties described above. Indeed, whereas a synthetic peptide composed of amino acids 197 to 216 and located within the HTLV-1 PRRH, has been reported to interfere with HTLV Env-induced syncytia formation [22], this peptide was later shown to compete neither with receptor binding of the entire HTLV-1 Env SU [38], nor with infection [26]. It is therefore likely that the effects reported for PRRH-derived peptides, as measured by syncytia formation, are solely due to post-receptor binding events. However, we identified Tyr_114 _of the HTLV-1 Env, which corresponds to Tyr_110 _of the HTLV-2 Env, as a key residue in HTLV Env binding and for all the aforementioned HTLV Env-mediated functional assays. We could not detect binding of H1(Y114A)SU by flow cytometry, while this mutant exerted residual, albeit significantly decreased, interference to HTLV Env-mediated cell fusion and infection. Altered folding outside of the binding domain *per se*, rather than direct alteration of the receptor-binding site, could also account for the lack of binding of this mutant. However, we favor the latter hypothesis, since the H1(Y114A)SU mutant was properly folded and transported to the plasma membrane and secreted in the medium as efficiently as wild type RBD, thus arguing against gross misfolding of this mutant. Accordingly, Tyr_114 _appears to be conserved in all known human and simian T cell leukemia viruses strains, which share the same receptor.

The receptor-binding site in MLV RBD is composed of a combination of several cysteine loops located upstream of the PRR [11,39] which is linked to a conserved anti-parallel β core [13]. The isolation of an F-MLV SU amino terminal subdomain allowed crystallization of MLV RBD and the modeling of the RBD cysteine loop arrangement [13]. The precise organization of cysteine loops, likely to harbor the receptor binding determinants, within the HTLV SU amino terminus remains to be established. Nevertheless, the identification of Tyr_114 _as a key HTLV-1 RBD residue points at this determinant as a very likely receptor-binding core. This, together with previous works relying on syncytia formation and cell-to-cell transmission [36,37], will help to distinguish between *bona fide *receptor binding determinants and determinants involved at a post-binding level.

Another recently identified determinant, the Pro-His-Gln SU motif conserved among gammaretroviruses such as MLV and feline leukemia viruses (FeLV), has been determined to play a major role in viral entry during post-binding events [40]. The mechanism of this effect involves a direct interaction of MLV SU soluble forms with Env attached SU carboxy terminus [41-46]. This interaction between the SU amino and carboxy termini leads to the T cell-restricted tropism of a natural isolate of FeLV, FeLV T, in which the SU Pro-His-Gln motif is mutated. Indeed, FeLV T is restricted in cat to T cells because they naturally express an endogenous soluble FeLV RBD-related factor called FeLIX that trans-complements the lack of the SU Pro-His-Gln motif in the FeLV T Env and restores its post-binding defect [47]. Despite the HTLV-1 and F-MLV SU homologous modular organization and the assignment of several common motifs between the two latter SU, no obvious Pro-His-Gln motif homologue is present in the HTLV SU amino terminus. Whether a FeLIX-like molecule that interacts with HTLV Env exists in human T cells remains to be addressed. Furthermore, the fact that the Pro-His-Gln has been shown to play a major role in transactivation of viral infection in several gammaretroviruses which are efficiently infectious as cell-free virions [42,44,48], raises the question whether the apparent lack of such a motif in the HTLV simple oncovirus-like SU is linked to the relative inefficiency of HTLV Env-mediated infection by cell-free virions. The HTLV SU subdomains described here should prove to be valuable in addressing such questions.

The recent identification of Glut1, the ubiquitous glucose transporter of vertebrates [49], as a receptor for HTLV Env [8] adds an additional similarity between the Env of HTLV, a deltaretrovirus, and that of gammaretroviruses. All these virus Env recognize multimembrane-spanning metabolite transporters [50,51]. This and the common modular organization of the HTLV and MLV SU raise questions regarding the origin of the HTLV Env. It has previously been reported that envelopes of invertebrate retroviruses may have been "captured" from other viruses [52-54]. As HTLV and MLV have strongly divergent overall genomic organizations, "envelope capture" from related ancestor genes might account for the close relationship between the Env of these phylogenetically distant viruses [10].

## Conclusions

We have generated truncated domains of the HTLV Env amino terminus, upstream of residues 183 and 178 of the HTLV-1 and -2 Env, respectively, that were sufficient to bind target cells of different species through interaction with the HTLV Env receptor. We also identified a tyrosine at position 114 and 110 in HTLV-1 and -2 Env, respectively, as a key determinant for this binding. In addition to their use for further exploration of the mechanisms involved in HTLV entry, the tagged HTLV-1 and -2 RBD subdomains described here are novel tools for the detection of Glut1 cell surface expression and intracellular trafficking. Indeed, we tracked intracellular expression of EGFP-tagged HTLV SU subdomains by time-lapse microscopy, and found that they are preferentially routed toward cell-cell contact areas (unpublished observations), where Glut1 is particularly abundant [55] and our unpublished observations). Furthermore, those HTLV SU derivatives could be of particular importance in view of the key roles played by Glut1 in various biological processes, including T cell survival and activation [31,56], tumor genesis [57,58], and neuronal activity [59]. Interestingly, soluble HTLV SU subdomains inhibit Glut1-mediated glucose transport, and accordingly, expression of mutants with diminished receptor binding ability resulted in less pronounced inhibition [8] and data not shown). Thus, these HTLV SU derivatives could also be used as glucose transport inhibitors. These data demonstrate the potential for the novel and broad utility of these reagents in the study of HTLV infection as well as biological processes involving glucose transport and metabolism.

## Materials and methods

### Construction of chimeric Env and HTLV-1 and -2 SU subdomains

To exchange the PRR and PRRH regions, we introduced an allelic *Mfe*I restriction site in the HTLV-1 and F-MLV Env. Introduction of this site in F-MLV resulted in the substitution of a glutamine and leucine (QL) dipeptide for the parental arginine and valine (RV) residues of the GPRVPIGP motif, at the start of the MLV Env PRR. Introduction of the *MfeI *site in the PSQL motif of the HTLV-1 SU maintained the parental QL residues, at the start of the HTLV Env PRRH. By exchanging domains at the *Mfe*I sites, we derived the H1_183_FEnv chimera containing the amino terminal 183 residues of the HTLV Env followed by the F-MLV PRR. In this chimera, the PSQL/PIGP hybrid sequence is generated at the exchange border, and the PRRH of HTLV is replaced by the F-MLV PRR (Figure 2A). In contrast, the entire PRRH of HTLV-1 is present in the H1_215_FEnv chimera – this Env chimera has been previously described and designated HHproFc [9]. The H1_183_FEnv and H1_215_FEnv chimeras, as well as the parental HTLV-1 and F-MLV Env, were inserted in an allelic fashion into the previously described pCEL retroviral Env expression vector [60]. The HTLV-2 Env expression vector, pCSIX/H2, was constructed by inserting the HindIII – EcoRI fragment from pHTE-2 (a gift from M-C Dokhelar) encompassing the HTLV-2 *env *gene, the pX region and the 3' LTR into pCSI (CMV promoter, SV-40 intron) [61] at the HindIII and EcoRI restriction sites.

The H1_215_SU, H2_211_SU, H1_179_SU, and H2_178_SU subdomains, corresponding to the HTLV-1 and -2 SU amino terminus with and without their respective PRRH, were generated by PCR and subcloned into the pCSI expression vector as fusion proteins harboring a carboxy terminal rFc or HA tag (Figure 2B). The H1(R94A)SU, H1(S101A)SU, H1(D106A)SU, and H1(Y114A)SU substitution mutants were generated by oligonucleotide-directed PCR mutagenesis on the H1_215_SU vector and subcloned into the pCSI expression vector. All PCR-generated DNA fragments were sequenced using an ABI Prism 310 sequencer. Cloning details are available upon request.

### Protein expression and immunoblots

Approximately 5 × 10^5 ^293T cells per 35 mm well were transfected with 5 μg of vectors using a calcium-phosphate-Hepes buffered saline (HBS) transfection protocol. Transfection medium was replaced with 3 ml of fresh culture medium twenty hours post-transfection. Forty-eight hours post-transfection cell culture medium (supernatant) was recovered and filtered through a 0.45 μm pore-size membrane to remove cell debris. Twenty μl were directly analyzed by SDS-PAGE (15% polyacrylamide gel), and the rest was aliquoted and stored at -20°C for later use in binding assays (see below). Cell extracts were collected 48 h post-transfection in 1 ml of cell lysis buffer (50 mM Tris-HCl [pH 8.0], 150 mM NaCl, 0.1% sodium dodecyl sulfate [SDS], 1% Nonidet P-40, 0.5% deoxycholate, and a cocktail of mammalian protease inhibitors [Sigma]) and clarified by two successive centrifugations at 13,000 rpm for 10 min at 4°C in a microcentrifuge. Approximately 20 μl of each extract, adjusted after normalization for protein concentration using the Bradford assay (Sigma), were subjected to electrophoresis on SDS-15% acrylamide gels, followed by transfer onto nitrocellulose (Protran; Schleicher & Schuell). Membranes were blocked in phosphate-buffered saline (PBS) containing 5% powdered milk and 0.5% Tween 20, probed with a 1:1000 dilution of a goat anti-RLV gp70 polyclonal antibody (Viromed) followed by a horseradish peroxidase-conjugated anti-goat immunoglobulin (for detection of chimeric Env), or goat anti-rabbit-IgG-horseradish peroxidase-conjugated immunoglobulins (for detection of rFc-tagged SU subdomains). Immunoblots were subsequently washed three times with PBS-0.1% Tween 20 and revealed by chemiluminescence (ECL+, Amersham).

### Binding and binding interference assays

Binding assays were performed as previously described [31]. Briefly, 5 × 10^5 ^target cells were detached with a PBS-EDTA solution, collected by centrifugation, incubated for 30' at 37°C with 300 μl of rabbit Fc-tagged soluble HTLV-1, HTLV-2, or Ampho-MLV truncated SU, washed, labeled with an anti-rabbit-IgG FITC-conjugated antibody, and analyzed on a FACSCalibur (Becton Dickinson). Data analysis was performed using the CellQuest software (Becton Dickinson). For interference studies, 293T cells were transfected with 4 μg of Env or Env SU subdomain expression vectors (carboxy terminal rFc-tagged forms) using the calcium-phosphate-HBS method. Under these conditions, transfection efficiencies ranged from approximately 80 to 90% of the target cells. Twenty-four and 48 hours post-transfection, cells were collected and transfected 293T cells expressing the different interfering HTLV or Ampho-MLV domains were incubated with a challenging HA-tagged soluble HTLV-2 SU amino terminal subdomain (H2_178_SU-HA). Cells were stained using a primary 12CA5 anti HA antibody followed by an anti-mouse-IgG FITC-conjugated antibody before detection by flow cytometry.

### Envelope interference to cell fusion assay

Briefly, the HTLV/MLV Env chimera, H1_183_FEnv, was used to interfere with challenging HTLV Env. The interfering non-fusogenic H1_183_FEnv and truncated HTLV SU subdomains were transiently transfected into HeLaCD4LTRLacZ, a cell line highly susceptible to HTLV Env-induced fusion that contains a stably integrated Tat-dependent LacZ expression vector [62]. These transfectants were cocultured with Tat-expressing NIH3T3(TK-) cells (NIH3T3(TK-)Tat) that were transiently transfected with the challenging HTLV Env. The NIH3T3(TK-)Tat cell line is resistant to HTLV-Env-induced syncytia formation, despite its ability to express the HTLV receptor and to bind HTLV Env, and thus can be used to precisely monitor fusion of the HeLaCD4LTRLacZ target cells [9,29]. H1_183_FEnv Env and truncated HTLV SU subdomains plasmid DNA (2 to 3 μg) was transfected into HeLaCD4LTRLacZ cells, while challenging, fusogenic HTLV-1 Env plasmid (1 μg) was transfected into NIH3T3(TK-)Tat. The interfering Env or SU subdomain-presenting cells were detached 24 hours post-transfection and 1–2 × 10^5 ^cells were cocultured for 24 hours with 1–2 × 10^5 ^challenging HTLV-1 Env-presenting NIH3T3(TK-)Tat cells. Subsequently, the cocultured cells were fixed and stained for β-galactosidase expression as described previously [60]. Transfection efficiencies of the HeLaCD4LTRLacZ target cells were approximately 50%. Mock transfections were performed with similar amounts of control plasmid DNAs. Env interference was measured by the decreased number of blue foci and was expressed as percent blue foci of control fusion (mock-transfected target cells). Data are represented as mean interference (± standard deviation), and statistical significance of interference levels was determined using a pairwise Student's *t *test.

### Envelope interference to infection assay

MLV(Ampho) and MLV(HTLV) pseudotyped virions were produced after transfection of 10^6 ^293T cells with 5 μg pCSI/Ampho or pCSIX/H2, respectively, 5 μg pCL/Gag-Pol [29] and 10 μg of pCLMFG-LacZ [63], using a calcium-phosphate-HBS transfection protocol. Supernatants were recovered 48 hours post transfection and filtered through 0.45 μm pore-size membrane to remove cell debris, and stored at -80°C. The pCLMFG-LacZ plasmid is a retroviral expression vector that provides a packageable RNA coding for the *LacZ *gene marker. pCSI/Ampho is an expression vector encoding the Ampho-MLV Env, and the HTLV-2 Env expression vector, pCSIX/H2, is described above.

Virion-containing supernatants were used to infect target 293T cells expressing the chimeric Env or HTLV RBD subdomains. Transfection efficiencies of target 293T cells were >80% in all experiments. Infections were performed 36–48 hours post-transfection on cultures grown in 12 well plates (Costar) at 37°C, medium was changed 24 hours later, and confluent cell monolayers were fixed, stained for β-galactosidase activity before counting blue foci. Interference to infection was determined by infecting transfected target cells with approximately 100 and 1000 iu. Infection was evaluated as described above, and the number of LacZ-positive blue colonies counted was normalized by multiplying by the appropriate dilution factor. The resulting infection values were analyzed as iu/ml of virus containing supernatant. Subsequently the relative infection levels in cells expressing the HTLV SU domains were compared to those of mock transfected cells and were expressed as percentages of control infection (% control).

## List of abbreviations used

HTLV Human T-cell leukemia virus

SU envelope extracellular surface component

Env envelope glycoprotein

MLV murine leukemia virus

F-MLV Friend-MLV

RBD receptor-binding domain

PRR proline-rich region

PRRH proline rich region homologue

Ampho amphotropic

HA influenza hemagglutinin

rFc rabbit immunoglobulin constant fragment

A_397_SU Ampho-MLV Env fused to a carboxy terminal rFc tag

CFIA cell fusion interference assay

iu/ml infectious units per ml

Arg_94_arginine 94

Ser_101_serine 101

Tyr_114_tyrosine 114

FeLV feline leukemia viruses

HBS Hepes buffered saline

PBS phosphate-buffered saline

SDS sodium dodecyl sulfate

## Competing interests

The authors declare that they have no competing interests.

## Authors' contributions

FJK designed and realized or supervised most of the experiments and co-wrote the manuscript. NM participated to some molecular constructions, set up, realized and analyzed most binding assays and FACS analyses and participated to the redaction of the manuscript. ENG set up and performed the cell-to-cell transmission assay and performed the corresponding experiments, CV constructed some of the RBD point mutants and tested them, MS initiated the project, co-participated in the design of the study, co-coordinated its realization and co-wrote the manuscript, and JLB realized some of the molecular constructs, performed some of the experiments, co-participated in the design of the study, co-coordinated its realization and co-wrote the manuscript. All authors read and approved the final manuscript.

## References

[B1] Richardson JH, Edwards AJ, Cruickshank JK, Rudge P, Dalgleish AG (1990). In vivo cellular tropism of human T-cell leukemia virus type 1. J Virol.

[B2] Hanon E, Stinchcombe JC, Saito M, Asquith BE, Taylor GP, Tanaka Y, Weber JN, Griffiths GM, Bangham CR (2000). Fratricide among CD8(+) T lymphocytes naturally infected with human T cell lymphotropic virus type I. Immunity.

[B3] Nagai M, Brennan MB, Sakai JA, Mora CA, Jacobson S (2001). CD8(+) T cells are an in vivo reservoir for human T-cell lymphotropic virus type I. Blood.

[B4] Wang TG, Ye J, Lairmore MD, Green PL (2000). In vitro cellular tropism of human T cell leukemia virus type 2. AIDS Res Hum Retroviruses.

[B5] Sutton RE, Littman DR (1996). Broad host range of human T-cell leukemia virus type 1 demonstrated with an improved pseudotyping system. J Virol.

[B6] Okuma K, Nakamura M, Nakano S, Niho Y, Matsuura Y (1999). Host range of human T-cell leukemia virus type I analyzed by a cell fusion-dependent reporter gene activation assay. Virology.

[B7] Trejo SR, Ratner L (2000). The HTLV receptor is a widely expressed protein. Virology.

[B8] Manel N, Kim FJ, Kinet S, Taylor N, Sitbon M, Battini JL (2003). The Ubiquitous Glucose Transporter GLUT-1 Is a Receptor for HTLV. Cell.

[B9] Kim FJ, Seiliez I, Denesvre C, Lavillette D, Cosset FL, Sitbon M (2000). Definition of an amino-terminal domain of the human T-cell leukemia virus type 1 envelope surface unit that extends the fusogenic range of an ecotropic murine leukemia virus. J Biol Chem.

[B10] Kim FJ, Manel N, Battini JL, Sitbon M (2004). Emergence of vertebrate retroviruses and envelope capture. Virology.

[B11] Battini JL, Heard JM, Danos O (1992). Receptor choice determinants in the envelope glycoproteins of amphotropic, xenotropic, and polytropic murine leukemia viruses. J Virol.

[B12] Battini JL, Danos O, Heard JM (1995). Receptor-binding domain of murine leukemia virus envelope glycoproteins. J Virol.

[B13] Fass D, Davey RA, Hamson CA, Kim PS, Cunningham JM, Berger JM (1997). Structure of a murine leukemia virus receptor-binding glycoprotein at 2.0 angstrom resolution. Science.

[B14] Koch W, Hunsmann G, Friedrich R (1983). Nucleotide sequence of the envelope gene of Friend murine leukemia virus. J Virol.

[B15] Gallaher WR, Ball JM, Garry RF, Martin-Amedee AM, Montelaro RC (1995). A general model for the surface glycoproteins of HIV and other retroviruses. AIDS Res Hum Retroviruses.

[B16] Sitbon M, d'Auriol L, Ellerbrok H, André C, Nishio J, Perryman S, Pozo F, Hayes SF, Wehrly K, Tambourin P, Galibert F, Chesebro B (1991). Substitution of leucine for isoleucine in a sequence highly conserved among retroviral envelope surface glycoproteins attenuates the lytic effect of the Friend murine leukemia virus. Proc Natl Acad Sci USA.

[B17] Andersen KB (1994). A domain of murine retrovirus surface protein gp70 mediates cell fusion, as shown in a novel SC-1 cell fusion system. J Virol.

[B18] Lavillette D, Maurice M, Roche C, Russell SJ, Sitbon M, Cosset FL (1998). A proline-rich motif downstream of the receptor binding domain modulates conformation and fusogenicity of murine retroviral envelopes. J Virol.

[B19] Tanaka Y, Zeng L, Shiraki H, Shida H, Tozawa H (1991). Identification of a neutralization epitope on the envelope gp46 antigen of human T cell leukemia virus type I and induction of neutralizing antibody by peptide immunization. J Immunol.

[B20] Tanaka Y, Tanaka R, Terada E, Koyanagi Y, Miyano-Kurosaki N, Yamamoto N, Baba E, Nakamura M, Shida H (1994). Induction of antibody responses that neutralize human T-cell leukemia virus type I infection in vitro and in vivo by peptide immunization. J Virol.

[B21] Londos-Gagliardi D, Jauvin V, Armengaut MH, Astier-Gin T, Goetz M, Huet S, Guillemain BJ (1999). Influence of amino acid substitutions on antigenicity of immunodominant regions of the HTLV type I envelope surface gylcoprotein: a study using monoclonal antibodies raised against relevant peptides. AIDS Res Hum Retroviruses.

[B22] Sagara Y, Inoue Y, Shiraki H, Jinno A, Hoshino H, Maeda Y (1996). Identification and mapping of functional domains on human T-cell lymphotropic virus type 1 envelope proteins by using synthetic peptides. J Virol.

[B23] Delamarre L, Pique C, Pham D, Tursz T, Dokhelar MC (1994). Identification of functional regions in the human T-cell leukemia virus type I SU glycoprotein. J Virol.

[B24] Delamarre L, Rosenberg AR, Pique C, Pham D, Callebaut I, Dokhelar MC (1996). The HTLV-I envelope glycoproteins: structure and functions. J Acquir Immune Defic Syndr Hum Retrovirol.

[B25] Jassal SR, Pohler RG, Brighty DW (2001). Human T-cell leukemia virus type 1 receptor expression among syncytium-resistant cell lines revealed by a novel surface glycoprotein-immunoadhesin. J Virol.

[B26] Jinno A, Haraguchi Y, Shiraki H, Hoshino H (1999). Inhibition of cell-free human T-cell leukemia virus type 1 infection at a postbinding step by the synthetic peptide derived from an ectodomain of the gp21 transmembrane glycoprotein. J Virol.

[B27] Chung M, Kizhatil K, Albritton LM, Gaulton GN (1999). Induction of syncytia by neuropathogenic murine leukemia viruses depends on receptor density, host cell determinants, and the intrinsic fusion potential of envelope protein. J Virol.

[B28] Siess DC, Kozak SL, Kabat D (1996). Exceptional fusogenicity of Chinese hamster ovary cells with murine retroviruses suggests roles for cellular factor(s) and receptor clusters in the membrane fusion process. J Virol.

[B29] Kim FJ, Manel N, Boublik Y, Battini JL, Sitbon M (2003). Human T-cell leukemia virus type 1 envelope-mediated syncytium formation can be activated in resistant Mammalian cell lines by a carboxy-terminal truncation of the envelope cytoplasmic domain. J Virol.

[B30] Ott D, Rein A (1992). Basis for receptor specificity of nonecotropic murine leukemia virus surface glycoprotein gp70SU. J Virol.

[B31] Manel N, Kinet S, Battini JL, Kim FJ, Taylor N, Sitbon M (2003). The HTLV receptor is an early T-cell activation marker whose expression requires de novo protein synthesis. Blood.

[B32] Sommerfelt MA, Weiss RA (1990). Receptor interference groups of 20 retroviruses plating on human cells. Virology.

[B33] Rein A (1982). Interference grouping of murine leukemia viruses: a distinct receptor for the MCF-recombinant viruses in mouse cells. Virology.

[B34] Rein A, Schultz A (1984). Different recombinant murine leukemia viruses use different cell surface receptors. Virology.

[B35] Chesebro B, Wehrly K (1985). Different murine cell lines manifest unique patterns of interference to superinfection by murine leukemia viruses. Virology.

[B36] Rosenberg AR, Delamarre L, Preira A, Dokhelar MC (1998). Analysis of functional conservation in the surface and transmembrane glycoprotein subunits of human T-cell leukemia virus type 1 (HTLV-1) and HTLV-2. J Virol.

[B37] Delamarre L, Rosenberg AR, Pique C, Pham D, Dokhelar MC (1997). A novel human T-leukemia virus type 1 cell-to-cell transmission assay permits definition of SU glycoprotein amino acids important for infectivity. J Virol.

[B38] Brighty DW, Jassal SR (2001). The synthetic peptide P-197 inhibits human T-cell leukemia virus type 1 envelope-mediated syncytium formation by a mechanism that is independent of Hsc70. J Virol.

[B39] Battini JL, Danos O, Heard JM (1998). Definition of a 14-amino-acid peptide essential for the interaction between the murine leukemia virus amphotropic envelope glycoprotein and its receptor. J Virol.

[B40] Bae Y, Kingsman SM, Kingsman AJ (1997). Functional dissection of the Moloney murine leukemia virus envelope protein gp70. J Virol.

[B41] Barnett AL, Cunningham JM (2001). Receptor binding transforms the surface subunit of the mammalian C-type retrovirus envelope protein from an inhibitor to an activator of fusion. J Virol.

[B42] Barnett AL, Davey RA, Cunningham JM (2001). Modular organization of the Friend murine leukemia virus envelope protein underlies the mechanism of infection. Proc Natl Acad Sci U S A.

[B43] Barnett AL, Wensel DL, Li W, Fass D, Cunningham JM (2003). Structure and mechanism of a coreceptor for infection by a pathogenic feline retrovirus. J Virol.

[B44] Lavillette D, Ruggieri A, Russell SJ, Cosset FL (2000). Activation of a cell entry pathway common to type C mammalian retroviruses by soluble envelope fragments. J Virol.

[B45] Lavillette D, Boson B, Russell SJ, Cosset FL (2001). Activation of Membrane Fusion by Murine Leukemia Viruses Is Controlled in cis or in trans by Interactions between the Receptor-Binding Domain and a Conserved Disulfide Loop of the Carboxy Terminus of the Surface Glycoprotein. J Virol.

[B46] Lavillette D, Ruggieri A, Boson B, Maurice M, Cosset FL (2002). Relationship between SU subdomains that regulate the receptor-mediated transition from the native (fusion-inhibited) to the fusion-active conformation of the murine leukemia virus glycoprotein. J Virol.

[B47] Anderson MM, Lauring AS, Burns CC, Overbaugh J (2000). Identification of a cellular cofactor required for infection by feline leukemia virus. Science.

[B48] Farrell KB, Ting YT, Eiden MV (2002). Fusion-defective gibbon ape leukemia virus vectors can be rescued by homologous but not heterologous soluble envelope proteins. J Virol.

[B49] Mueckler M, Hresko RC, Sato M (1997). Structure, function and biosynthesis of GLUT1. Biochem Soc Trans.

[B50] Overbaugh J, Miller AD, Eiden MV (2001). Receptors and entry cofactors for retroviruses include single and multiple transmembrane-spanning proteins as well as newly described glycophosphatidylinositol-anchored and secreted proteins. Microbiol Mol Biol Rev.

[B51] Tailor CS, Lavillette D, Marin M, Kabat D (2003). Cell surface receptors for gammaretroviruses. Curr Top Microbiol Immunol.

[B52] Malik HS, Henikoff S, Eickbush TH (2000). Poised for contagion: evolutionary origins of the infectious abilities of invertebrate retroviruses. Genome Res.

[B53] Rohrmann GF, Karplus PA (2001). Relatedness of baculovirus and gypsy retrotransposon envelope proteins. BMC Evol Biol.

[B54] Pearson MN, Rohrmann GF (2002). Transfer, Incorporation, and Substitution of Envelope Fusion Proteins among Members of the Baculoviridae, Orthomyxoviridae, and Metaviridae (Insect Retrovirus) Families. J Virol.

[B55] Virgintino D, Robertson D, Benagiano V, Errede M, Bertossi M, Ambrosi G, Roncali L (2000). Immunogold cytochemistry of the blood-brain barrier glucose transporter GLUT1 and endogenous albumin in the developing human brain. Brain Res Dev Brain Res.

[B56] Rathmell JC, Vander Heiden MG, Harris MH, Frauwirth KA, Thompson CB (2000). In the absence of extrinsic signals, nutrient utilization by lymphocytes is insufficient to maintain either cell size or viability. Mol Cell.

[B57] Smith TA (1999). Facilitative glucose transporter expression in human cancer tissue. Br J Biomed Sci.

[B58] Younes M, Lechago LV, Somoano JR, Mosharaf M, Lechago J (1996). Wide expression of the human erythrocyte glucose transporter Glut1 in human cancers. Cancer Res.

[B59] Magistretti PJ, Pellerin L (2000). [Functional brain imaging: role metabolic coupling between astrocytes and neurons]. Rev Med Suisse Romande.

[B60] Denesvre C, Sonigo P, Corbin A, Ellerbrok H, Sitbon M (1995). Influence of transmembrane domains on the fusogenic abilities of human and murine leukemia retrovirus envelopes. J Virol.

[B61] Battini JL, Rasko JE, Miller AD (1999). A human cell-surface receptor for xenotropic and polytropic murine leukemia viruses: possible role in G protein-coupled signal transduction. Proc Natl Acad Sci U S A.

[B62] Dragic T, Charneau P, Clavel F, Alizon M (1992). Complementation of murine cells for human immunodeficiency virus envelope/CD4-mediated fusion in human/murine heterokaryons. J Virol.

[B63] Naviaux RK, Costanzi E, Haas M, Verma IM (1996). The pCL vector system: rapid production of helper-free, high-titer, recombinant retroviruses. J Virol.

[B64] Ragheb JA, Anderson WF (1994). pH-independent murine leukemia virus ecotropic envelope-mediated cell fusion: implications for the role of the R peptide and p12E TM in viral entry. J Virol.

[B65] Rein A, Mirro J, Haynes JG, Ernst SM, Nagashima K (1994). Function of the cytoplasmic domain of a retroviral transmembrane protein: p15E-p2E cleavage activates the membrane fusion capability of the murine leukemia virus Env protein. J Virol.

